# Protein–protein interaction analysis reveals a novel cancer stem cell related target TMEM17 in colorectal cancer

**DOI:** 10.1186/s12935-021-01794-2

**Published:** 2021-02-06

**Authors:** Zhao-liang Yu, Yu-feng Chen, Bin Zheng, Ze-rong Cai, Yi-feng Zou, Jia Ke, Ping Lan, Feng Gao, Xiao-jian

**Affiliations:** 1 Department of Colorectal Surgery, The Sixth Affiliated Hospital of Sun Yat-Sen University, Guangzhou, Guangdong China; 2 Guangdong Provincial Key Laboratory of Colorectal and Pelvic Floor Diseases, The Sixth Affiliated Hospital of Sun Yat-Sen University, 26 Yuancun Erheng Rd, Guangzhou, 510655 Guangdong China; 3 Guangdong Institute of Gastroenterology, Guangzhou, China

**Keywords:** Colorectal cancer, Cancer stem cell, TMEM17, Chemoresistance, Protein–protein interaction

## Abstract

**Background:**

Cancer stem cells (CSCs) are a small subpopulation of cells within tumors with stem cell property. Increased evidence suggest that CSCs could be responsible for chemoresistance and recurrence in colorectal cancer (CRC). However, a reliable therapeutic target on CSCs is still lacking.

**Methods:**

Here we describe a two-step strategy to generate CSC targets with high selectivity for colon stem cell markers, specific proteins that are interacted with CSC markers were selected and subsequently validated in a survival analysis. TMEM17 protein was found and its biological functions in CRC cells were further examined. Finally, we utilized the Gene Set Enrichment Analysis (GSEA) to investigate the potential mechanisms of TMEM17 in CRC.

**Results:**

By combining protein–protein interaction (PPI) database and high-throughput gene profiles, network analysis revealed a cluster of colon CSCs related genes. In the cluster, *TMEM17* was identified as a novel CSCs related gene. The results of in-vitro functional study demonstrated that *TMEM17* depletion can suppress the proliferation of CRC cells and sensitize CRC cells to chemotherapy drugs. Enrichment analysis revealed that the expression of TMEM17 is associated with the magnitude of activation of the Wnt/β-catenin pathway. Further validation in clinical samples demonstrated that the TMEM17 expression was much higher in tumor than normal tissue and was associated with poor survival in CRC patients.

**Conclusion:**

Collectively, our finding unveils the critical role of TMEM17 in CRC and TMEM17 could be a potential effective therapeutic target for tumor recurrence and chemoresistance in the colorectal cancer (CRC).

## Introduction

Colorectal cancer (CRC) is one of the most common cancer types in the world. A recent statistic study demonstrated that over one million new cases of CRC were identified in 2018 globally [1]. Although advances in the treatments of CRC improved the disease outcome, the tumor recurrence and chemoresistance remain major causes of therapy failure [2, 3]. A highly treatment resistant core portion of cancer cells, termed “cancer stem cells” (CSCs), could be responsible for these treatment failures. In line with this hypothesis, studies have discovered stem cell markers including LGR5, CD24, CD44, EPCAM, CD133 on a small proportion of CRC population [4–11]. In addition, this highly chemotherapy resistant population of CSCs exhibits stem cell properties including self-renewal and generation of mature differentiated cancer cells [12, 13]. These findings suggest that the CSC population could be a potential target for CRC treatment, its application value in clinic need to be examined.

Since abundant amount of patients’ genomic profiles has been taken up in public data sets, which are free of access, the high-throughput data analysis has emerged as a new efficient and cost saving method for the cancer research [14]. In this study, we found a cluster of cancer stem cell related proteins using a systematic and protein interaction network analysis. Among these proteins, TMEM17 is closely related to the colon cancer stem cell markers. TMEM17 belongs to the transmembrane (TMEM) protein family, which is involved in numerous pathological processes of the cancer development, such as local invasion, metastasis formation and intravasation [15, 16]. Diverse functions of TMEM17 family member have been reported, for example TMEM48, TMEM45A and TMEM97 were reported as potential prognostic biomarkers for cancers, TMEM16A was found related to calcium regulation and TMEM173 was reported to control the immune response in carcinogenesis [16–18].

To our knowledge, just a few studies have shown that TMEM17 is associated with the cancer development. One claimed that TMEM17 is a pro-oncogenic protein in the breast cancer, while another declared that TMEM17 is an anti-oncogenic protein in the lung cancer [19, 20]. It is still unknown why TMEM17 demonstrated these contradict effects and what is its effect on other type of cancers such as CRC. In this study, we describe a two-step strategy to generate CSC targets with high selectivity for colon stem cell markers, specific proteins associated with CSC markers were selected and subsequently validated in a survival analysis. TMEM17 was found in the selecting process and its biological functions was further investigated in CRC cell lines. The results demonstrated that depletion of TMEM17 enhances the sensitivity of chemotherapy drugs and suppresses the Wnt/β-catenin signaling. Taken together, our study identifies an important role of TMEM17 in colon cancer and elucidates a potential cancer stem cell target to sensitize chemotherapy.

## Methods

### Patients

We retrospectively analyzed the gene expression profiles of frozen colorectal cancer tumor tissue samples from one of the largest individual data sets: CIT/GSE39582 CRC cohort. The data set was obtained directly in its processed format from GEO database through Bioconductor package ‘GEOquery’. All patients were included in this study. The batch effects were corrected using ‘combat’ algorithm implemented in R package ‘sva’ and z-scores for each gene were used for the following analyses. Both paper charts and electronic medical records were carefully reviewed when necessary.

### Construction and analysis of protein–protein interaction network

To find potential therapeutic targets on colon CSCs, nine colon stem cell markers selected from previous studies were used to construct a protein–protein interaction (PPI) network related to colon CSCs (Table 1). The protein interaction information of these proteins were obtained from the BioGRID database (Version 3.5.168) [21, 22]. To investigate the association of chemotherapeutic sensitivity, 232 patients with chemotherapy and complete prognostic information in the CIT cohort (GSE39582) were used as the discovery data set [23]. To obtain genes related to prognosis of colorectal cancer and avoid the influence of sample distribution, the corresponding genes resulted from PPI analysis were further examined using the log-rank test with 1000 times randomization (80% portion of samples each time) to assess the association between each gene and patients’ disease-free survival in the discovery cohort. Genes with significant frequency found in more than 500 times in repeated log-rank tests were identified as key genes.

**Table 1:** List of colon cancer stem cell markers

Gene	Other name	Function	References
Lgr5	GPR49	Wnt signaling gene	[4, 8, 38–40]
ALDH1A1	ALDC, ALDH1	Enzyme	[7]
CD24	CD24A	Cell adhesion molecule	[8]
CD29	Integrin b1	Cell adhesion molecule	[8]
CD44	CDW44	Cell adhesion molecule, Hyaluronic acid receptor	[5, 8, 9]
CD133	Prominin 1	Self-renewal, Tumor angiogenesis	[6, 8]
CD166	ALCAM	Cell adhesion molecule	[8]
EPCAM	ESA, MK-1	Cell adhesion molecule	[9]
MSI1	Musashi-1	RNA-binding protein	[10, 11]

### Validation cohort

The CIT cohort, one of the largest individual data sets of colorectal cancer was used to validate the potential therapeutic value of TMEM17. The expression of TMEM17 were analysed in 17 cancer samples and its paired normal tissue, while other 566 patients’ data were used to conduct a prognostic analysis. The optimal cut-off point of TMEM17 expression was determined based on disease-free survival (DFS) information of these patients using the function 'surv_cutpoint' from R package 'survminer'.

### Short interfering RNA

The sequence of used *TMEM17* siRNAs are si*TMEM17* #1: GCAGCATTATGAT GCTTCA; si*TMEM17* #3: GGTCATGTATAGAAGAGAT. The Lipofectamine RNAiMAX kit (Invitrogen) was used for siRNA transfection following the manufacturer’s instructions. Cells were transfected with 100 nM final concentration of siRNA duplexes at the optimal seeding density. After 24 h, cells were re-seeded for following experiments.

### RT-quantitative PCR

Total RNA was isolated using TRIzol reagent (Life Technologies, Carlsbad, CA) and the RNeasy Mini Kit (Qiagen, Germany), and subsequently reverse transcribed into cDNA using the cDNA Synthesis Kit (Transgen Biotech, China). RT-PCR was performed using the KAPA SYBR Fast qPCR kit (KAPA Biosystems, Wilmington, MA). For quantification of mRNA levels, *18S* level was used as an internal control. The specific primers used for *TMEM17* were: 5′-GTTCAGTGATTCCAATCGGACC- 3′; 3′- ACCACAGTGGGAA ATAGTAGGT-5’.

### Immunoblotting

Cells were collected and lysed in RIPA buffer supplemented with protease and phosphatase inhibitors for 30 min. Equal amounts of protein extract were separated on SDS polyacrylamide gels and transferred to polyvinyl difluoride (PVDF) membranes. Membranes were blocked with 5% BSA for 2 h at room temperature and then probed with primary antibody overnight at 4 °C. The used antibodies were anti-TMEM17 (Santa Cruz, CA; sc-514433), anti-EPCAM (Beyotime, China; AF0141), anti-LGR5 (Abcam, UK; ab75850), anti-MYC (Abcam, UK; ab32072), anti-Vimentin (Cell Signaling Technology, USA; #5741), anti-Snail (Cell Signaling Technology, USA; #3879) and anti-GAPDH (Cell Signaling Technology, USA; #5174).

### Cell proliferation assay

To conduct the cell proliferation assay, optimal cells were plated in triplicate in a 96-well format. After 24 h, the medium was refreshed with optimal drug treatment. Cells were then lysed with CellTilter-Glo (CTG, Promega, Madison, WI), and the fluorescence signal was detected with a microplate reader on days 0, 2, 3, 4 and 5.

### Colony formation assay and Tumorsphere formation assay

To conduct the clonogenic assay, optimal cells were seeded in 6 well plates and refresh the medium every 3 days in 37℃. Colonies were formed after 8 to 10 days culture. The colonies were fixed with methanol and stained with crystal violet (0.5% crystal violet, 20% methanol).

To conduct the tumorsphere assay, single-cell suspensions were plated (5000 cells/well) in a 12 well ultra-low attachment plates with Mammocult medium (Stem cell Technologies), which is supplemented with fresh hydrocortisone (0.5 μg/ml) and heparin (1:500) and culture in a 37 ℃ 5%CO_2_ incubator. Medium was refreshed every 3 day and tumorspheres were formed after 7–10 days culture. The spheres were isolated and stained with 2-(4-iodophenyl)-3-(4-nitrophenyl) 5-phenyl-2H-tetrazolium chloride (INT) (Sigma-Aldrich) and quantified.

To generate the oxaliplatin-resistant cells, DLD1 cells were grown in medium supplemented with increasing concentration of oxaliplatin during the culture period. The concentration of oxaliplatin in the medium increased every three passages until the cells were totally resistant to it. The Oxaliplatin-resistant cells were verified by clonogenic assay.

### Plasmid constructs and constructed cell lines

Full-length TMEM17 was amplified by RT-PCR using 100 ng total RNA from HEK293 cells and the following primers: 5′-ATGGAGCTGCCGGATCCGGT-3′ and 5′-TCAGATCTCTTCTATACATG-3′. The PCR product was cloned into pCMV/GFP for overexpression studies. After selected with puromycin (5 µg/ml), the generated clones were screened for experiments.

### Pathway analysis

Enrichment analysis was performed for differentially expressed genes between high and low TMEM17 expressing groups using R package ‘gProfileR’ in the CIT data set. The cut-off point of high/low TMEM17 expression was determined based on the optimal cut-off point in disease-free survival (DFS) analysis in the CIT. For interested biological pathways, Gene Set Enrichment Analysis (GSEA) was further performed using Bioconductor package ‘HTSanalyzeR’ [24, 25].

### Tissue microarray and immunohistochemistry staining

A total of 318 CRC patients with pTNM stage I to III from January 2002 to June 2006 were included in this study and the pathological specimens were constructed in a tissue microarray (TMA). Tumor staging was assessed according to the criteria of the Seventh Edition of the American Joint Committee on Cancer (AJCC) stage [26]. The clinicopathological data were collected from the CRC database of the follow-up office and approved by the Institutional Review Board of the The Sixth Affiliated Hospital, Sun Yat-sen University.

Paraffin-embedded tissue samples were cut into 5 μm sections and antigen retrieval was performed with citrate buffer (Beyotime, China; P0081). After blocking with 10% goat serum, samples were incubation with primary antibody overnight at 4 °C, followed by diaminobenzidine staining. IHC staining was evaluated in semi-quantitative method as described before [27]. Each TMA spot was marked with an intensity score and percentage of positive tumor cells was scored from 1 to 4. TMA scores were determined by the intensity score multiply proportion of area score. A final score was calculated as the average of the duplex. The optimal cut-off point of TMEM17 expression was conducted based on X-tile software (X-tile 3.6.1) [28].

### Statistical analysis

Graphs were expressed as *mean* ± *SD* from three independent experiments. Statistical difference between two groups was evaluated by two-tailed student’s *t*-test, or by two-way ANOVA for multiple groups. Survival curves were evaluated by Log-rank (Mantel-Cox) test. *P*-values < 0.05 were considered as statistically significant.

## Result

### Protein–protein interaction network analysis identified TMEM17 as a CSC related marker

**Fig. 1:**
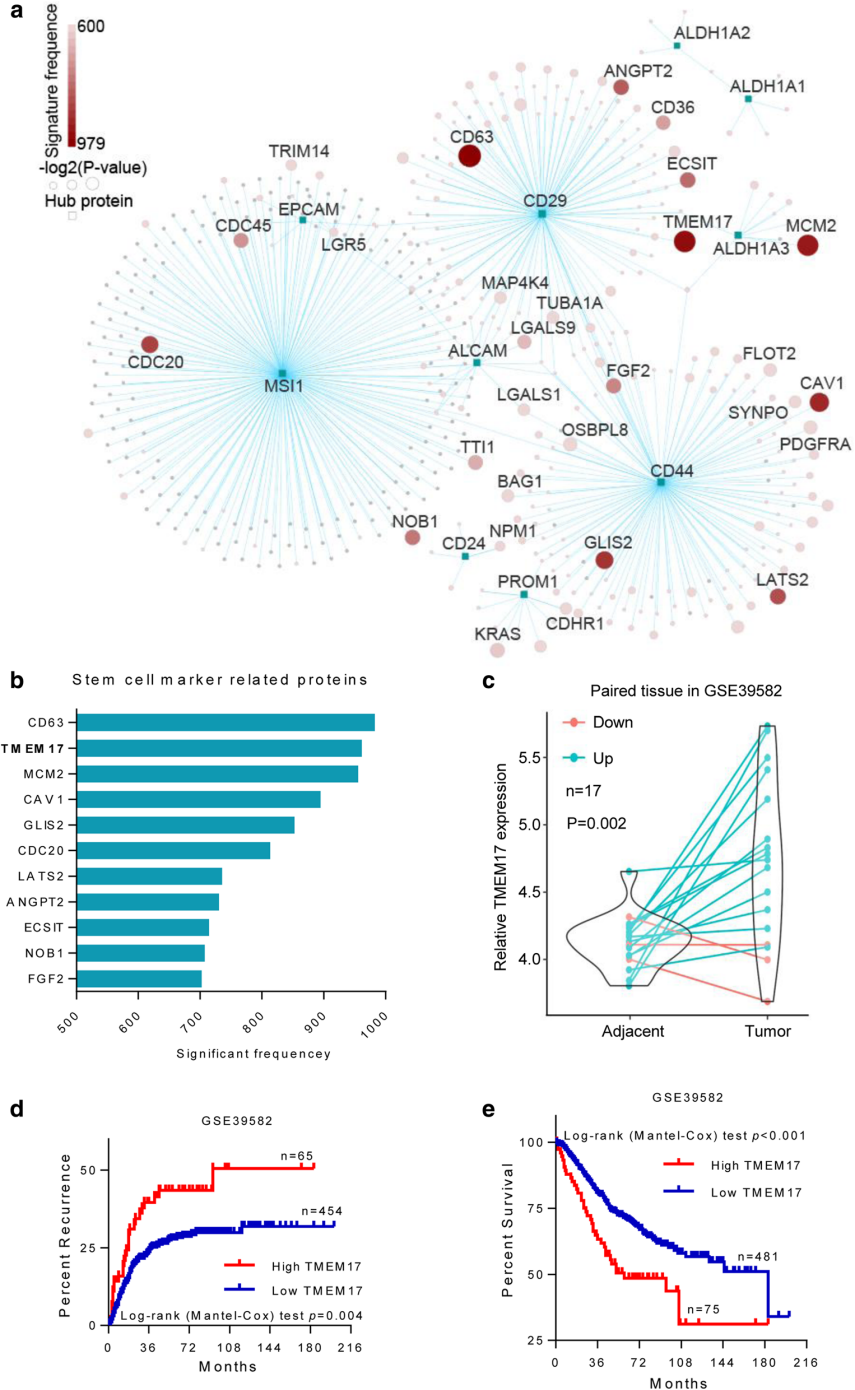
PPI network and survival analysis identified *TMEM17* as a CSC related gene. **a** PPI network of the nine colon stem cell markers. Node size is -log2 transformed averaged *P*-values in 1000 randomization log-rank tests. Node color represents the frequency calculated by the number of times that the corresponding gene significantly associated with survival in the same analysis. Nodes with labels represent key genes related to CSC (frequency > 500). Edges represent physical PPIs between proteins obtained from BioGRID database. **b** The significant frequency of 11 CSC related genes in survival analysis (log-rank test, *P* < 0.05; frequency > 500). **c** *TMEM17* expression is significantly higher in CRC samples than that in paired normal colon tissue (*P* = 0.002). **d**, **e** Kaplan–Meier survival analysis revealed that high *TMEM17* expression was significantly correlated with tumor recurrence (**d**) and short overall survival (**e**)

To find potential therapeutic targets on colon CSCs, a PPI network was constructed based on a list of colon stem cell markers (Fig. 1a, Table 1). A total of 683 records were found using the PPI analysis, containing 276 different proteins. The corresponding genes of these 276 proteins were verified using log-rank test to evaluate the relationship between each gene and patients’ diseases free survival in the CIT data set. Eleven genes were identified and listed based on the significant frequency in the resampling survival analyses and further filtered by the average *P* value (Fig. 1b, Table 2). Among these genes, the function of *TMEM17* in the development of CSC is unknown and its effect on the development of CRC has not been reported yet. Analysing of the mRNA expression of TMEM17, an increased expression was found in the tumor tissue as compare to adjacent normal tissue (Fig. 1c). In addition, increased *TMEM17* expression was associated with tumor recurrence and poor survival (Fig. 1d, e). This result indicated that it could be a novel biomarker to predict CRC prognosis.

**Table 2:** Stem cell marker related proteins

Gene	Full name	Frequency in resampling
CD63	CD63 molecule	979
TMEM17	Transmembrane protein 17	958
MCM2	Minichromosomal maintenance complex component 2	952
CAV1	Caveolin 1	891
GLIS2	GLIS family zinc finger 2	849
CDC20	Cell division cycle20	810
LATS2	Large tumor suppressor kinase 2	732
ANGPT2	Angiopoietin 2	727
ECSIT	ECSIT signalling integrator	711
NOB1	NIN1 (RPN12) binding protein 1 homolog	704
FGF2	Fibroblast growth factor 2	699

### Genetic depleting TMEM17 supressed cell proliferation in CRC

**Fig. 2:**
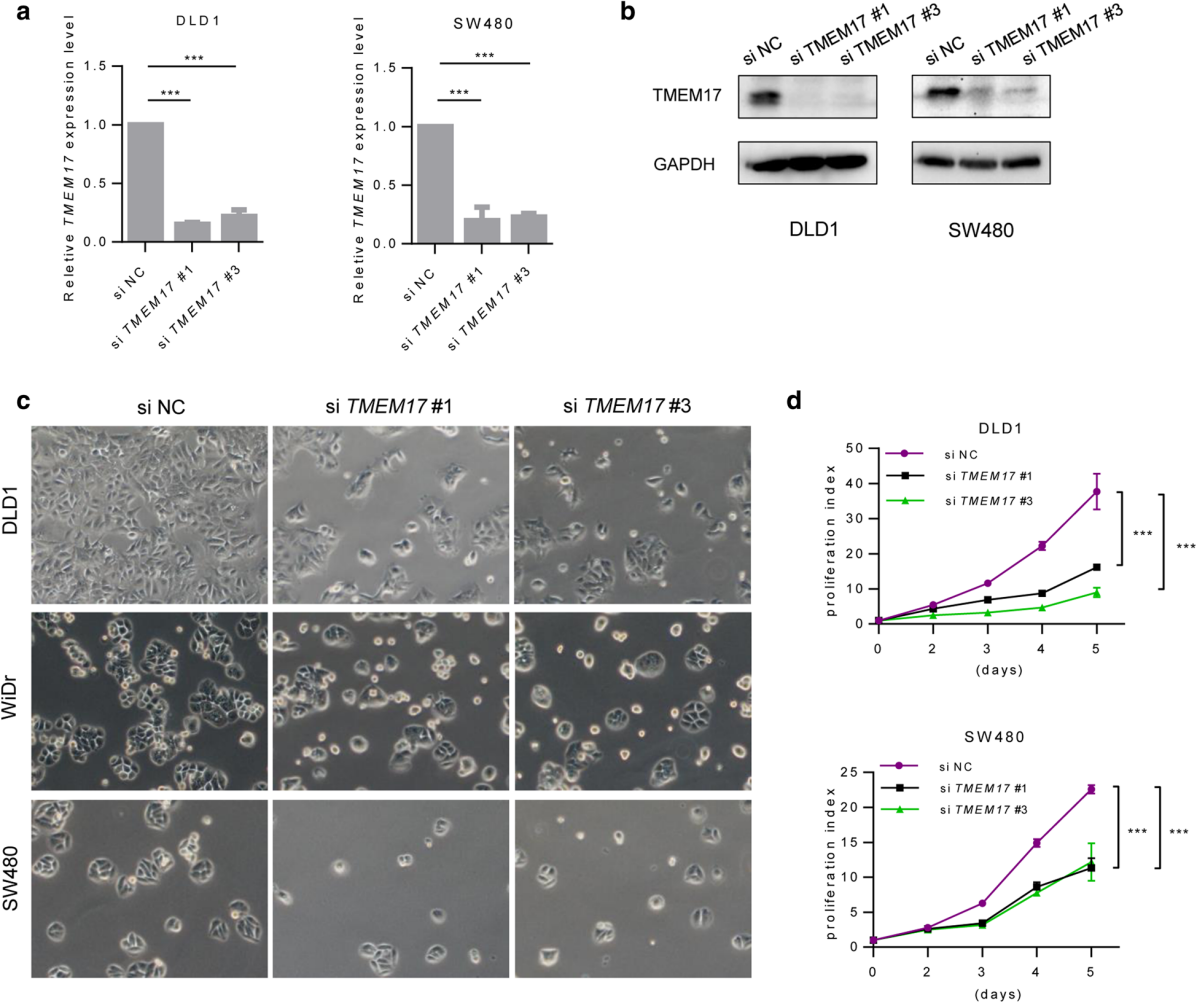
Depletion of TMEM17 suppressed CRC cells proliferation. **a**, **b** The transfection efficiency after depleting TMEM17 by siRNA in CRC cells were tested by RT-PCR (**a**) and immunoblotting (**b**). **c** Representative images of 48 h after CRC cells treated with si-TMEM17 in adherent culture. The bar = 200um. **d** Growth curves of CRC cells with depleting TMEM17 in a period of 5 days culture. *p < 0.05, **p < 0.01, ***p < 0.001, student’s T test (**a**), one-way ANOVA (**d**), as compared to the control group

To investigate TMEM17 biological function in cancer cells, we detected TMEM17 expression in a panel of CRC cell lines (Additional file 1: Figure S1) and selected high expressed cell lines for genetic depletion by using siRNA. The depletion effect was conformed using RT-qPCR and immunoblotting assay (Fig. 2a, b). Significantly reduced colony formation and cell proliferation were found when cells were transfected with *TMEM17* siRNA (Fig. 2c, d). To investigate biological function of TMEM17 in normal cells, two normal colon mucosa cell lines (NCM460 and HIEC6) were selected to genetic deplete of TMEM17 by siRNA. The results show that suppress TMEM17 expression hardly have any affect to proliferation of normal colon mucosa cells (Additional file 1: Figure S1B–D). These results suggest that TMEM17 plays as a crucial role in colon cancer cell proliferation and it may be a potential therapeutic target of CRC.

### Targeting TMEM17 enhanced the sensitivity of chemotherapy drugs in CRC

**Fig. 3:**
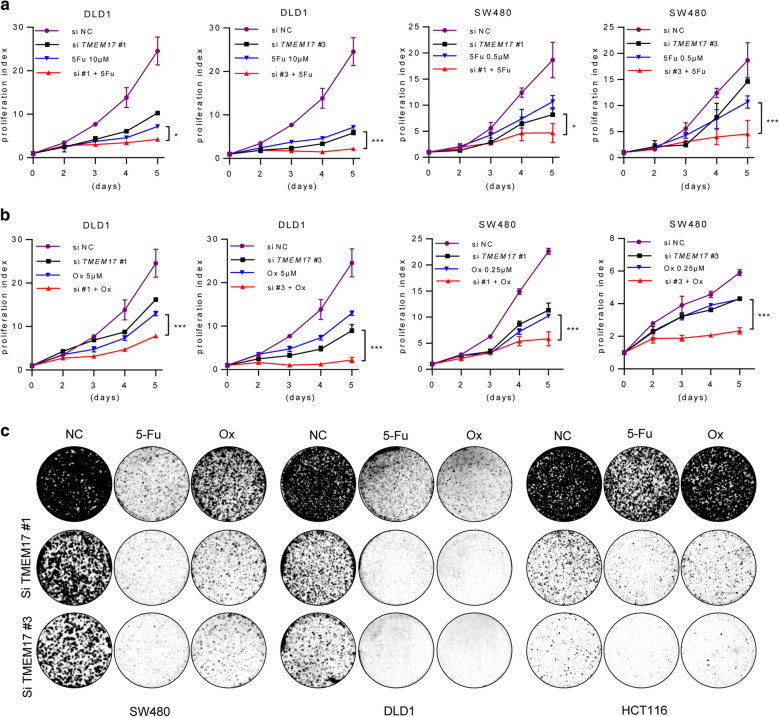
Targeting TMEM17 enhanced the sensitivity of chemotherapy drugs in CRC. **a** Growth curves of CRC cells with depleting TMEM17 and 5-Fu treatment in a period of 5 days culture. **b** Growth curves of CRC cells with depleting TMEM17 and oxaliplatin treatment in a period of 5 days culture. **c** Clonogenic assay of CRC cells with depleting TMEM17 and 5-Fu/oxaliplatin treatment in a period of 8 to 10 days culture. *p < 0.05, **p < 0.01, ***p < 0.001, two-way ANOVA (**a**)

Chemotherapy failure is one of crucial reasons for the tumor recurrence in stages II–III CRC patients. Hence, enhancing the sensitivity of chemotherapy drugs is a potential therapeutic strategy for CRC treatment. To investigate the potential pharmacology value of TMEM17, cells were treated with chemotherapy drugs after siRNA transfection. The cell proliferation rate was significantly suppressed in TMEM17 siRNA transfected cells treated with anti-tumor drugs as comparing to the cells receive the drug treatment alone. The choose of the drug, neither 5-Fu or oxaliplatin treatment affected this outcome (Fig. 3a, b). In line with these findings, si-TMEM17 enhanced the sensitivity of 5-Fu and oxaliplatin treatment in a long-term cell culture system (Fig. 3c).

### Targeting TMEM17 suppressed CSC characteristic in CRC cells

**Fig. 4:**
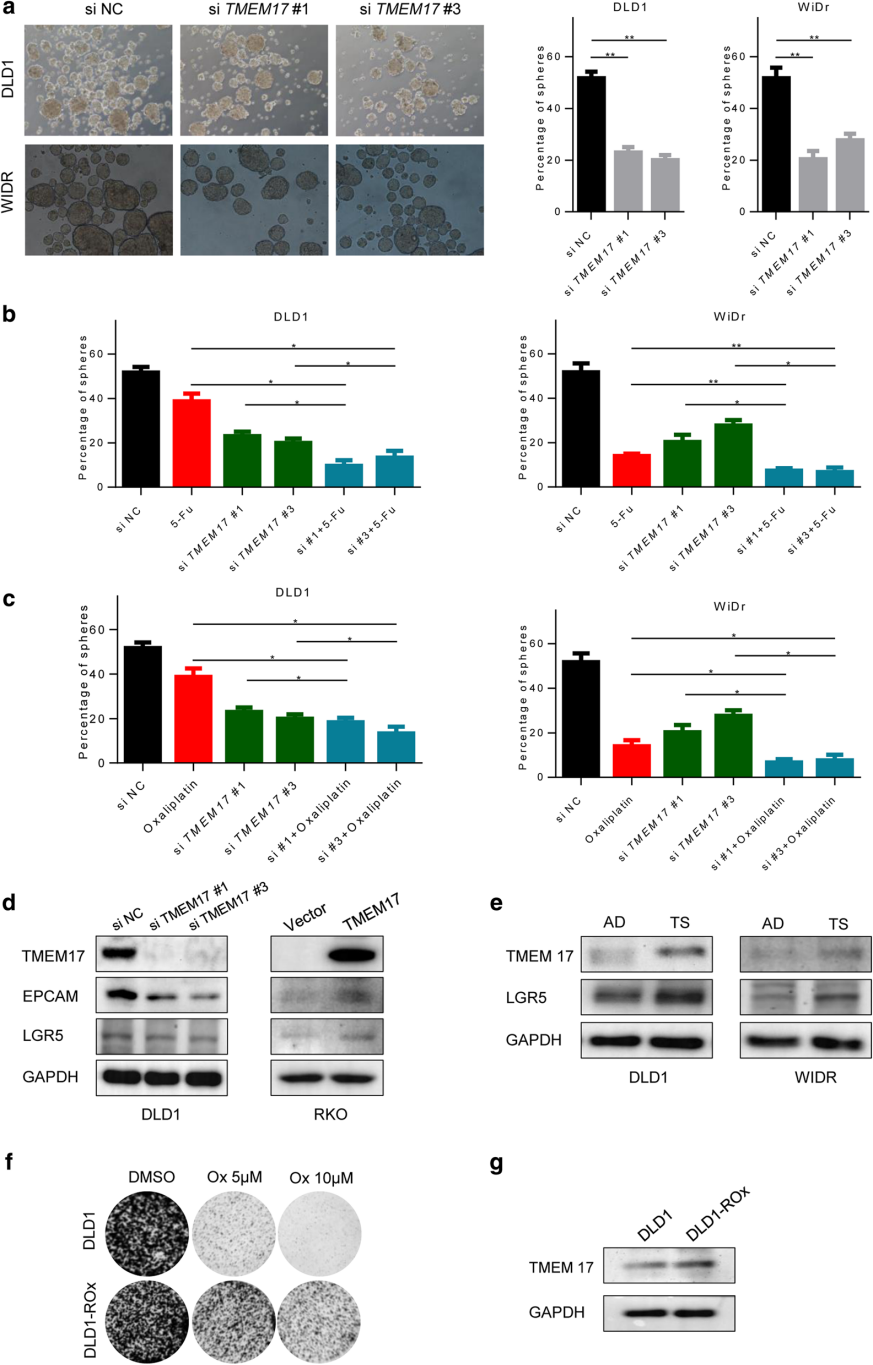
Targeting TMEM17 suppressed CSC characteristic in CRC cells. **a** Genic depleting TMEM17 reduced cancer cell sphere formation, n = 3. The bar = 400um. **b** Tumorsphere formation assay of CRC cells with depleting TMEM17 and 5-Fu treatment, n = 3. **c** Tumorsphere formation assay of CRC cells with depleting TMEM17 and Oxaliplation treatment, n = 3. **d** Immunoblotting assay of the expression of TMEM17, EPCAM and LGR5 proteins from CRC cells with scramble or TMEM siRNA. (Left) Immunoblotting assay of the expression of TMEM17, EPCAM and LGR5 proteins from CRC cells with vector or TMEM17. (Right) **e** Immunoblotting assay of the expression of TMEM17, EPCAM and LGR5 proteins from CRC cells with adherent culture or tumorsphere culture. **f** Clonogenic assay of DLD1 and DLD1 oxaliplatin resistance cells. **g** Immunoblotting assay of DLD1 and DLD1 oxaliplatin resistance cells. Error bars represent ± SD. **P < 0.01, *P < 0.05, paired sample T test (**a**–**c**)

To test the functional importance of TMEM17 in CSC, colon cancer cells were cultured in serum free medium to examine sphere formation. Depleting of TMEM17 significantly suppressed the spheres formation (Fig. 4a) and enhanced the sensitivity of chemotherapy drugs (Fig. 4b, c). Notably, the CSC markers, including EPCAM and LGR5, were downregulated in si-TMEM17 transfected cells and were upregulated after over expressing TMEM17 in RKO cells (Fig. 4d). We also investigated the expression of TMEM17 when cells were cultured in different culture mediums. TMEM17 was increased in tumorsphere culture, accompanying with CSC markers up-expression (Fig. 4e). One of well-known chemoresistance hypothesis is that the CSCs escape from the chemotherapy drug killing and form new drug resistant cancer cell population [29, 30]. In line with this hypothesis, TMEM17 was upregulated in DLD1 oxaliplatin resistance cells. This observation indicated that TMEM17 may be an importance factor during cells switching from drug sensitivity to resistance. Together, these findings show that the expression of TMEM17 is associated with the colon CSCs development and targeting TMEM17 may help enhance chemotherapy efficiency.

### TMEM17 related CSC features were associated with Wnt/ β-catenin signaling

**Fig. 5:**
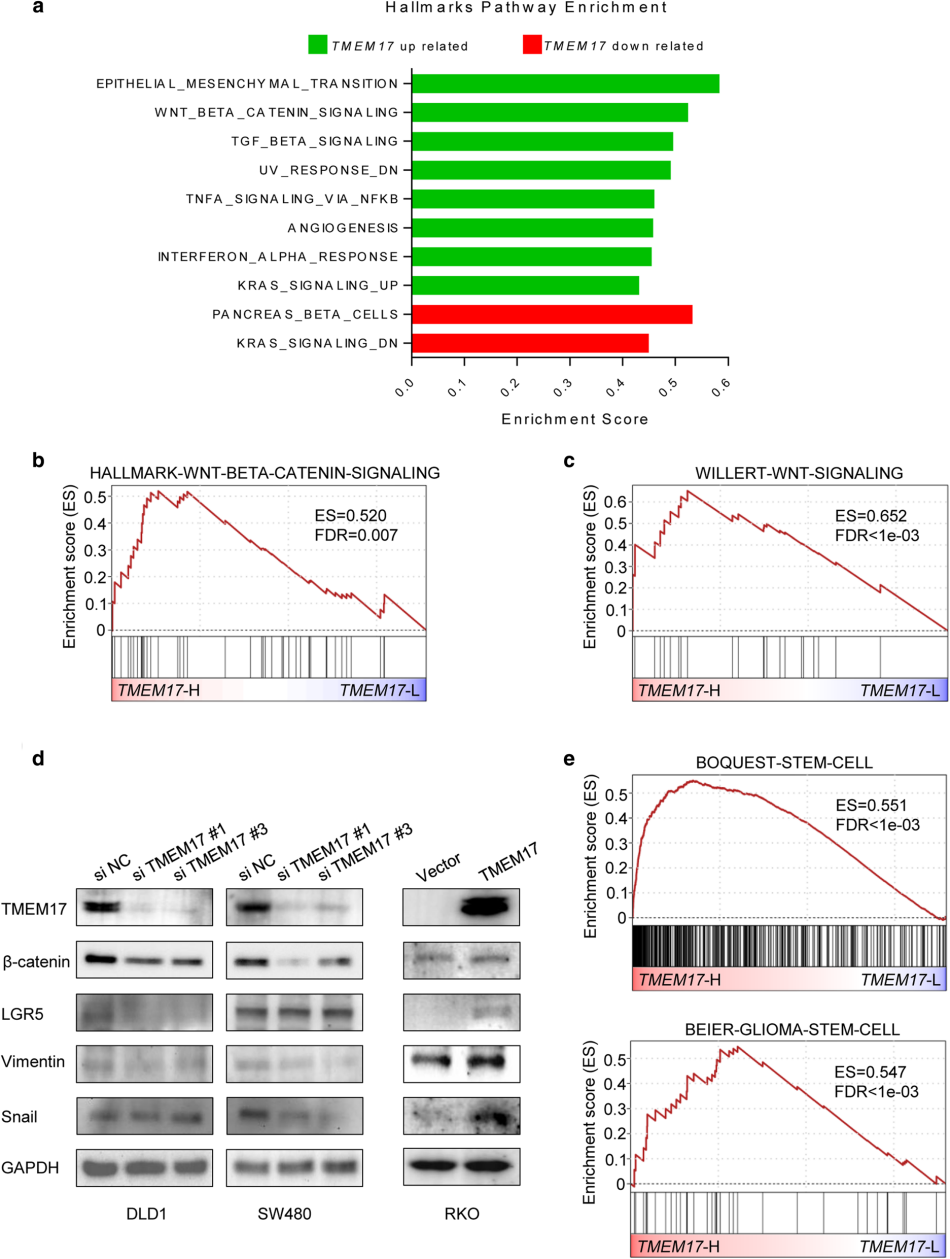
Pathway enrichment analysis between differential expression groups of TMEM17. **a** Significantly dysregulated pathways were identified by GSEA in the CIT cohort for cancer hallmark pathways. Top ten pathways were selected for presentation by absolute enrichment score (positive score is green and negative score is red). **b** GSEA plot of the Hallmarks Wnt/ β-catenin signaling in the CIT cohort. **c** GSEA plot of Willert Wnt signalling in the CIT cohort. **d** Immunoblotting assay of the expression of TMEM17 and several Wnt signaling markers proteins from CRC cells with scramble or TMEM siRNA. (Left) Immunoblotting assay of the expression of TMEM17 and several Wnt signaling markers proteins from CRC cells with vector or TMEM17. (Right) **e** GSEA plot of Boquest Stem Cell signalling and Beier Glioma Stem Cell signalling in the CIT cohort

In order to find the underlying mechanism of TMEM17 mediated CSC development, pathway analysis was performed with patients’ genomic profiles in the CIT data set. The result suggested that high *TMEM17* expression was associated with active epithelial mesenchymal transition, Wnt/β-catenin signaling and TGF β signaling (Fig. 5a, Additional file 2: Table S1). Among these signaling pathways, the Wnt/β-catenin signaling, which is known to regulate the activation and differentiation of tumor initiating cells [31–34], was enrichment in high *TMEM17* patients (Fig. 5a–c). The results of the immunoblotting analysis have demonstrated that depletion of TMEM17 suppressed some makers of Wnt/β-catenin signaling (Fig. 5d). Thus, these markers were upregulated after over expressing TMEM17 in RKO cells (Fig. 5d). In addition, patients with the high *TMEM17* expression have shown a significant enrichment of stem cell gene sets (Fig. 5e). These data suggest that *TMEM17* may be a positive regulator of the development of CSCs.

### TMEM17 is upregulated in CRC and is related to poor CRC survival

**Fig. 6:**
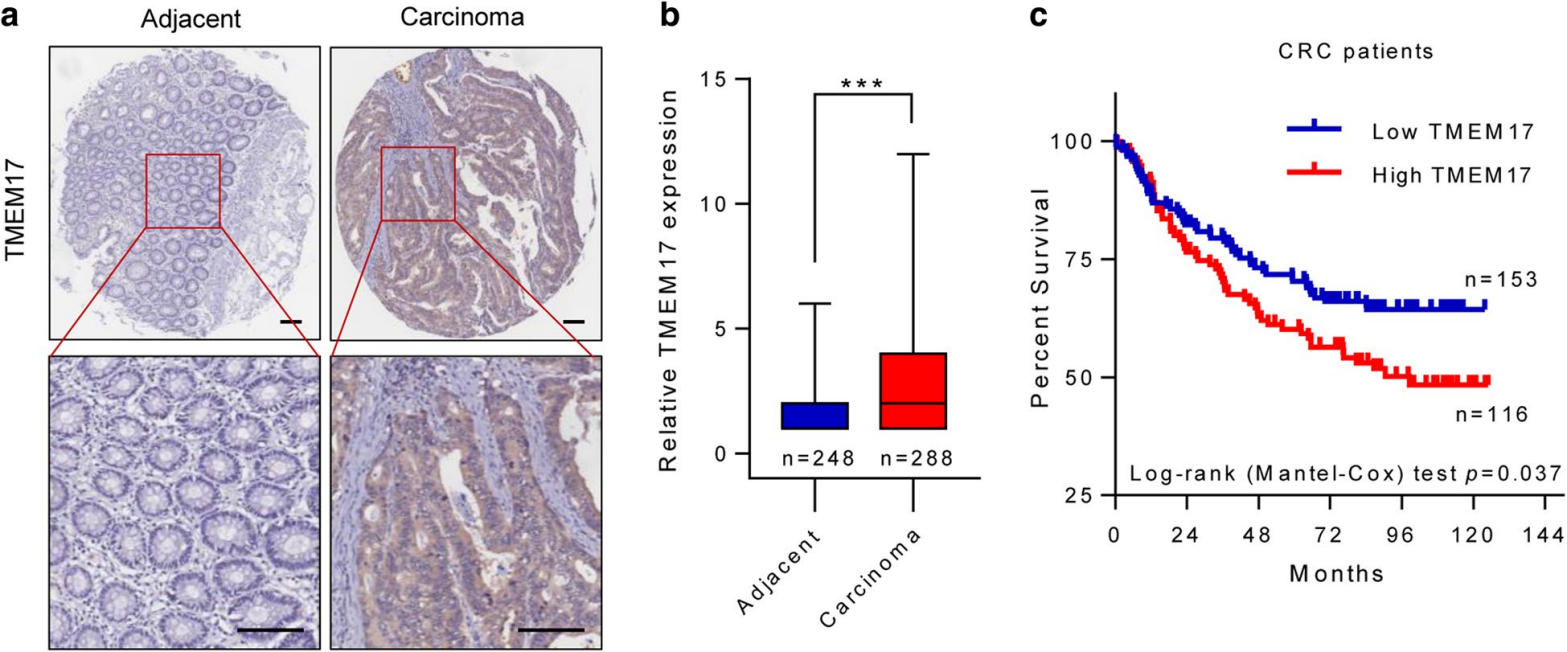
TMEM17 is upregulated in CRC and is related to poor CRC survival. **a** Representative TMEM17 immunochemistry staining in CRC and normal colon tissue. The scale bar represents 100 μm. **b** Tissue microarray assay of TMEM17 expression in CRC and normal colon tissue. Error bars represent ± SD. ***P < 0.001, 2-tailed unpaired Student’s t test. **c** High TMEM17 expression was associated with poor overall survival in CRC patients

Based on the functional studies of TMEM17, we hypothesized that TMEM17 activity may affect the clinical outcome of CRC patients. We constructed a tissue microarray containing a large cohort of CRC patients and tested the expression of TMEM17 (Fig. 6a, Additional file 3: Table S2). An increased expression of TMEM17 was found in tumor tissue as compared to the normal tissue. In addition, TMEM17-high group was associated with a significant lower survival rate as compared to the TMEM17-low group (Fig. 6b, c). The survival analysis revealed a 10-year survival rate of 64% in TMEM17-low group, while 48% in TMEM17-high group (P = 0.037).

## Discussion

CSCs are believed to be highly chemoresistance and play a key role as tumor initiating cells in cancer recurrence following chemotherapy [35]. However, there is no effective CSCs-targeting strategy in the clinical use, the main reason is no valid target on CSCs has been confirmed yet [36, 37]. This study is aimed to find a potential therapeutic target on CSCs using the big data analysis method. A cluster of potential target protein was found combining the protein–protein interaction (PPI) database and high-throughput gene profiles. Among these proteins, the TMEM protein 17 was selected and further investigated for its biological functions in CRC cells.

The existence of CSCs was proposed decades ago. Although increasing studies find evidence support the CSCs theory, their effort still face a fundamental scepticism that many still doubt if CSCs is a distinct tumor cell population and whether CSCs are involved in the recurrence of every cancer types [37]. In CRC, a series of lineage-tracing studies confirmed that a LGR5^+^ cell population had the ability to undergo differentiation in different models [38–40]. These studies supporting that CRC is composed of heterogeneous cell populations including a small fraction of CSC. Currently, most of anti-CSCs strategies were based on targeting the stem cell markers or inhibiting the relevant pathway signalling, such as anti-CSCs antigens and Wnt inhibitors [41]. In this study, we performed a PPI analysis using selected CSC markers and discovered that TMEM17 may as a potential target on CSCs.

Genome-scale human PPI networks are useful systems that can help find clusters of genes from the same features and provide more potential targets for the cancer therapy [42]. PPI network analysis was shown to be a reliable tool to interpret the function of abundant genes associated with the development of cancer and autism [42]. Increasing studies using PPI analysis in cancer research indicate that it is a potential method to discover new therapeutic targets [43, 44]. Here, we constructed a PPI network using nine colon CSC markers and discovered a cluster of significant targets. Most of these genes were related to CSCs, such as *CD63* [45], *MCM2* [46], *CAV1* [47], *GLIS2* [48], *CDC20* [49, 50], *LATS2* [51] and *FGF2* [52, 53]. Interestingly, *TMEM17*, being the marker with the second highest resampling frequency in the survival analysis, has not been reported as marker of CSCs yet and its pathological function in other tumors has been found to be contradicted [19, 20]. Hence, it’s deserved to be investigated.

PPI analysis is a promising method for the discovery of therapeutic target due to its high efficiency, but it also subjects to the limitations of the bioinformatics analysis. Even though we have conducted series of in vitro experiments to validate the pathological function of TMEM17, these limitations could not be totally ignored. Firstly, although we analysis the prognostic information of public database and used a large cohort of CRC patient samples, in vitro experiments using primary paired tissue samples were lacking. Secondly, our results reveal that depletion of TMEM17 may inhibit the proliferation of CSCs by suppressing the Wnt/β-catenin signaling, but the underlying mechanisms are still unclear and need to be clarified in the future.

In conclusion, we performed a PPI analysis based on colon CSC makers and discovered a novel CSC related gene *TMEM17*. Investigating the biological function of TMEM17 in CRC cells, we found that TMEM17 may be contribute to the proliferation of the CSC population within the CRC cells. These data suggest that TMEM17 could be a potential effective therapeutic target for tumor recurrence and chemoresistance in the colorectal cancer (CRC).

## Supplementary Information

**Additional file 1: Figure S1. **(**A**) Immunoblotting assay of the expression of TMEM17 in a panel of CRC cells. (**B**) Immunoblotting assay of the expression of TMEM17 after depleting TMEM17 by siRNA in normal colon mucosa cells. (**C**) Representative images of 48 h after normal colon mucosa cells treated with si-TMEM17 in adherent culture. (**D**) Clonogenic assay of CRC cells with depleting TMEM17 in a period of 8 to 10 days culture.

**Additional file 2: Table S1. **The HALLMARK pathway analysis of patients’ genomic profiles in the CIT data set.

**Additional file 3: Table S2. **Patient characteristics of the tissue microarray database.

## Data Availability

The data sets generated and analyzed during the current study are available in the GEO database (CIT/GSE39582, https://www.ncbi.nlm.nih.gov/geo/query/acc.cgi?acc=GSE39582) and Genomic Data Commons Data Portal (TCGA CRC, https://portal.gdc.cancer.gov/).
