# Correction to: Protein–protein interaction analysis reveals a novel cancer stem cell related target TMEM17 in colorectal cancer

**DOI:** 10.1186/s12935-021-01877-0

**Published:** 2021-03-18

**Authors:** Zhao‑liang Yu, Yu‑feng Chen, Bin Zheng, Ze-rong Cai, Yi‑feng Zou, Jia Ke, Ping Lan, Feng Gao, Xiao‑jian Wu

**Affiliations:** 1 Department of Colorectal Surgery, The Sixth Affiliated Hospital of Sun Yat-Sen University, Guangzhou, Guangdong China; 2 Guangdong Provincial Key Laboratory of Colorectal and Pelvic Floor Diseases, The Sixth Affiliated Hospital of Sun Yat-Sen University, 26 Yuancun Erheng Rd, Guangzhou, 510655 Guangdong China; 3 Guangdong Institute of Gastroenterology, Guangzhou, China

## Correction to: Cancer Cell Int (2021) 21:94 https://doi.org/10.1186/s12935-021-01794-2

Following publication of the original article [1], we were notified of a mistake in the corresponding author’s name.

- Originally published name: Xiao-jian
- Corrected name: Xiao-jian Wu

The original article has been corrected.
