# Supplementary figures and images for: Protein–protein interaction analysis reveals a novel cancer stem cell related target TMEM17 in colorectal cancer
Source: Cancer Cell Int. 2021 Feb 6;21:94. doi: 10.1186/s12935-021-01794-2 (PMC7868027; doi:10.1186/s12935-021-01794-2)

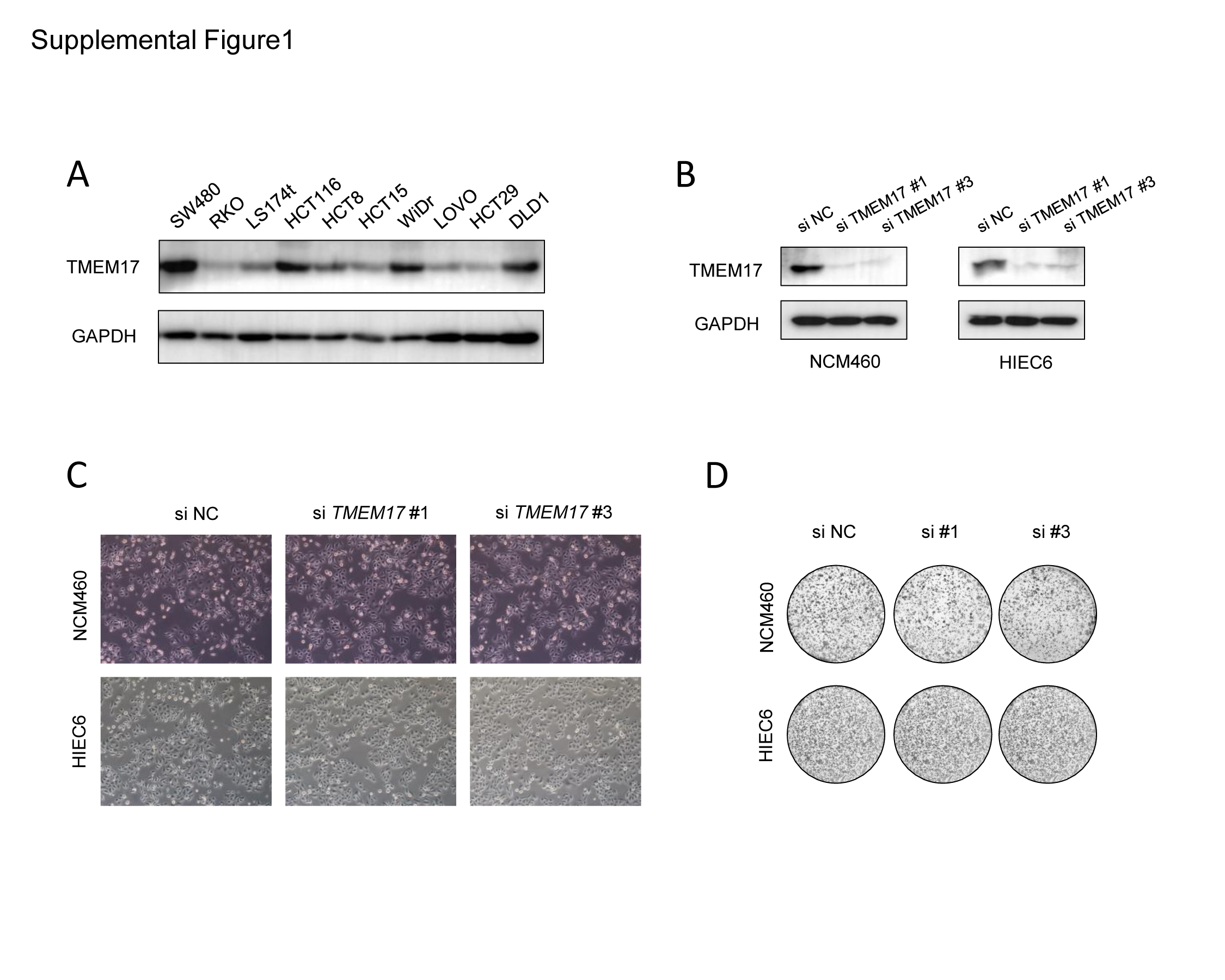

Supplement: Supplementary file 1 — Additional file 1: Figure S1. (A) Immunoblotting assay of the expression of TMEM17 in a panel of CRC cells. (B) Immunoblotting assay of the expression of TMEM17 after depleting TMEM17 by siRNA in normal colon mucosa cells. (C) Representative images of 48 h after normal colon mucosa cells treated with si-TMEM17 in adherent culture. (D) Clonogenic assay of CRC cells with depleting TMEM17 in a period of 8 to 10 days culture. [file 12935_2021_1794_MOESM1_ESM.tif]
