# Supplementary material for: Protein–protein interaction analysis reveals a novel cancer stem cell related target TMEM17 in colorectal cancer
Source: Cancer Cell Int. 2021 Feb 6;21:94. doi: 10.1186/s12935-021-01794-2 (PMC7868027; doi:10.1186/s12935-021-01794-2)
Supplement: Supplementary file 3 — Additional file 3: Table S2. Patient characteristics of the tissue microarray database. [file 12935_2021_1794_MOESM3_ESM.docx]

**Table S2.** Patient characteristics of the tissue microarray database

| Characteristic | All cases |
| --- | --- |
| Number of patients | 318 |
| Age at the time of surgery, yrs | 59.38± 11.48 |
| Male patients, n (%) | 175 (55.0%) |
| Pathological T stage, n (%) |  |
| pT0 or pT1 or pT2 | 48 (15.1%) |
| pT3 or pT4 | 270 (84.9%) |
| Pathological N stage, n (%) |  |
| pN0 | 181 (56.9%) |
| pN1 or pN2 | 137 (43.1%) |
| Pathological M stage, n (%) |  |
| pM0 | 318 (100%) |
| Pathological TNM stage, n (%) |  |
| pTNM0 or pTNM1 or pTNM2 | 180 (56.6%) |
| pTNM3 or pTNM4 | 138 (43.4%) |
| Histopathology, n (%) |  |
| Adenocarcinoma | 315 (99.1%) |
| Others | 3 (0.9%) |
| Differentiation, n (%) |  |
| Well | 94 (29.8%) |
| Moderate or poor | 221 (70.2%) |
| Median follow-up time, yrs | 6.32 |
